# Hydroxypropyl methylcellulose stearoxy ether hydrogel loaded with *aloe vera* peel-derived extracellular vesicle mimetics promotes wound healing in diabetic mice

**DOI:** 10.3389/fbioe.2026.1768930

**Published:** 2026-03-25

**Authors:** Jiatong Wang, Peng Wu, Juan Hu, YiLin Wang, Tianci Lang, Yue Tan, Ge Yu, Shizhu Cai, Ruihan Liu, Yichi Sun, Nan Liu, Fang Wang, Linghe Zang, Dongchun Liu

**Affiliations:** 1 School of Chinese Materia Medica, Shenyang Pharmaceutical University, Shenyang, China; 2 Joint International Research Laboratory of Intelligent Drug Delivery Systems of Ministry of Education, Shen-yang Pharmaceutical University, Shenyang, China; 3 Hainan Enneas Technology Co., Ltd., Haikou, China; 4 School of Pharmacy, Shenyang Pharmaceutical University, Shenyang, China; 5 Faculty of Functional and Wine, Shenyang Pharmaceutical University, Shenyang, China; 6 School of Clinical Pharmacy, Shenyang Pharmaceutical University, Shenyang, China

**Keywords:** aloe vera, diabetic wound healing, extracellular vesicle mimetics, hydrogel delivery system, hydroxypropyl methylcellulose stearoxy ether, Sangelose

## Abstract

**Background:**

The impaired healing of diabetic wounds under hyperglycemic conditions presents a major challenge. While *Aloe vera* gel is known for wound healing, the potential of its peel is underexplored. This study aimed to develop a scalable production method for *Aloe vera* peel-derived extracellular vesicle mimetics (AVp-EVMs), screen an optimal hydrogel for their delivery, and evaluate their efficacy in diabetic wound healing.

**Methods:**

AVp-EVMs were produced via high-pressure homogenization and ultracentrifugation. Their effects on keratinocyte (HaCaT) proliferation/migration and anti-inflammatory activity in LPS-stimulated macrophages were assessed *in vitro*. Two hydrogels (non-ionic Sangelose 60L and ionic Carbomer 940) were screened as carriers. Finally, AVp-EVMs and the optimal hydrogel formulation (HG-EVM) were evaluated in a diabetic mouse wound model by monitoring wound closure, histology, and immunohistochemistry.

**Results:**

AVp-EVMs showed good biocompatibility, promoted keratinocyte proliferation and migration, and were effectively internalized. They suppressed LPS-induced inflammation in macrophages by inhibiting NF-κB activation and downstream pro-inflammatory cytokines (TNF-α, IL-1β, IL-6). The Sangelose hydrogel preserved the AVp-EVMs structure better than the Carbomer hydrogel. In diabetic mice, both AVp-EVMs and HG-EVM dressing promoted wound healing by enhancing re-epithelialization, reducing inflammation, and improving collagen deposition and alignment. The HG-EVM group achieved the highest wound closure rate (97.2%), significantly outperforming free AVp-EVMs injection (94.9%; *P* < 0.01).

**Conclusion:**

This study establishes a production method for AVp-EVMs. The non-ionic Sangelose hydrogel is an effective delivery vehicle, and the HG-EVM formulation enhances diabetic wound healing more effectively than free AVp-EVMs alone.

## Graphical Abstract

**Figure:**
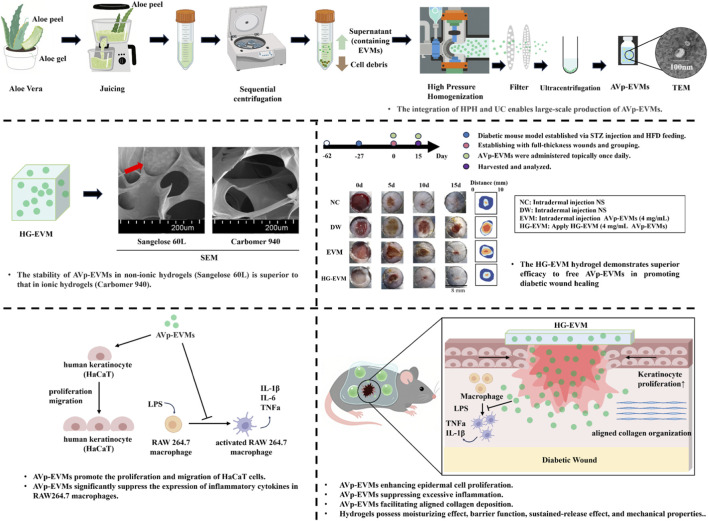

## 1 Introduction

Impaired wound healing is one of the most intractable complications of diabetes, globally affecting approximately 15% of diabetic patients (Shou et al., 2023; Wu et al., 2024). Its pathological complexity primarily stems from the accumulation of advanced glycation end products induced by persistent hyperglycemia, which disrupts collagen cross-linking and inhibits cellular proliferation and migration (Liu et al., 2019). Concurrently, the dominance of M1 macrophages in the wound microenvironment leads to excessive cytokine release (e.g., Tumor Necrosis Factor-α, TNF-α; Interleukin-1 beta, IL-1β), further impeding tissue repair (Castleberry et al., 2016). Current clinical strategies, including debridement, negative pressure wound therapy, and growth factor application, exhibit limited efficacy, with recurrence rates reaching 65%, underscoring the urgent need for efficient therapeutic approaches (Yoon et al., 2016; Chen et al., 2017).

In recent years, plant-derived extracellular vesicles (PEVs) have demonstrated considerable potential in dermatology due to their good biocompatibility, biomimetic lipid structure, and notable ability to penetrate the skin barrier (Narauskaitė et al., 2021; Wu et al., 2023). Studies have shown that PEVs can carry various active components—such as miRNAs, antioxidants, and anti-inflammatory compounds—and deliver them to target cells, thereby exhibiting therapeutic promise in areas including skin aging, abnormal pigmentation, hair loss, and wound healing (Ju et al., 2013; Narauskaitė et al., 2021; Anusha and Priya, 2022; Kyung and Lee, 2023; Mu et al., 2023; Wu et al., 2023; Liu et al., 2025). PEVs are composed of a phospholipid bilayer membrane and exhibit distinctive lipid characteristics. These include high levels of phosphatidic acid, phosphatidylcholine, digalactosyl diacylglycerol, and monogalactosyl diacylglycerol, which differentiate them from other vesicle-based systems. In addition, PEVs contain a variety of proteins that function as channels and transporters within the membrane. This unique composition confers natural PEVs with superior biocompatibility, stability, *in vivo* distribution, prolonged half-life, and enhanced cellular internalization (Mu et al., 2023).

*Aloe vera*, a traditional medicinal plant, plays a significant role in promoting skin repair and wound healing. Although *Aloe vera* peel-derived EVs have demonstrated multiple potential functions—such as promoting cell migration, exerting antioxidant and anti-inflammatory effects, and inhibiting excessive activation of myofibroblasts—in various cellular models, research progress has been constrained by conventional extraction techniques (Kim et al., 2021; Ramírez et al., 2024). Current strategies, such as tissue dispersion combined with ultracentrifugation, primarily target the isolation of extracellular vesicles. However, these methods are associated with low extraction yields and fail to efficiently utilize intracellular vesicle components. As a result, functional validation of these vesicles remains largely confined to the cellular level (Konoshenko et al., 2018; Kim and Park, 2022; Wang R. et al., 2025).

To overcome these limitations, researchers have explored various physical extraction strategies. For instance, Abraham et al. employed ultracentrifugation and high-pressure homogenization, respectively, to extract vesicles from cucumbers and found that vesicles obtained via the latter method further enhanced drug penetration efficiency through the skin, highlighting the potential of physical disruption methods for preparing functional plant vesicles (Abraham et al., 2022). According to the guidelines issued by the MISEV Organizing Committee, such EV-like particles (EVPs) obtained through direct cell disruption are referred to as EV mimetics (EVMs) (Welsh et al., 2024). Inspired by this approach, the present study introduces, for the first time, a combined method of high-pressure homogenization and ultracentrifugation to isolate *aloe vera* peel-derived EV mimetics (AVp-EVMs). This strategy first employs high-pressure homogenization to disrupt plant cell structures, facilitating the release of intracellular vesicles and further reducing vesicle size, thereby generating a mixture of intra- and extracellular vesicle components. Subsequent ultracentrifugation is used to concentrate the homogenate, which is expected to shorten centrifugation time, reduce vesicle aggregation, and improve overall yield.

In wound treatment, topical application and periwound subcutaneous injection are two common drug administration methods. However, within the complex wound microenvironment, exosomes administered solely via topical application are susceptible to washout by bodily fluids, making it difficult to maintain an effective concentration. Although periwound subcutaneous injection can increase the initial drug concentration around the wound and avoid rapid loss, it still faces challenges such as a relatively high systemic clearance rate and the need for frequent injections, which may lead to decreased patient compliance (Qiu et al., 2025). Hydrogel, as a novel dressing and delivery system with a three-dimensional cross-linked network structure, demonstrates significant advantages as a drug carrier due to its good swelling properties, extracellular matrix-mimetic functions, and controllable release capabilities (Wang Z. et al., 2025). Currently, numerous studies focus on single administration routes for exosomes, whereas systematic comparisons of the therapeutic efficacy among different delivery strategies remain scarce. Based on this, the present study established a full-thickness skin wound model in diabetic mice to systematically compare the effects of topical application of hydrogel loaded with AVp-EVMs (HG-EVM) and periwound subcutaneous injection of AVp-EVMs solution on promoting wound healing, aiming to comprehensively evaluate their therapeutic potential.

## 2 Materials and methods

### 2.1 Materials

#### 2.1.1 Plant material

*Aloe vera* L. was provided by Hainan Jiumiantong Technology Co., Ltd. (Haikou, China).

#### 2.1.2 Cell lines

Human immortalized keratinocytes (HaCaT), which were obtained from Sunncell Biotechnology Co., Ltd. (Wuhan, China), were used to validate the proliferative and migratory effects of AVp-EVMs on skin cells.

Murine macrophages (RAW264.7), sourced from Shanghai Zhongqiao Xinzhuo Biotechnology Co., Ltd. (Shanghai, China), were employed to assess the anti-inflammatory activity of AVp-EVMs against lipopolysaccharide (LPS)-induced inflammation.

All cell lines were cultured in Dulbecco’s Modified Eagle Medium (DMEM, Sigma-Aldrich, US) supplemented with 10% fetal bovine serum (Gibco, Grand Island, NY) and 1% penicillin-streptomycin (Sigma-Aldrich, US) at 37 °C in a humidified incubator under 5% CO_2_. Experiments were conducted at 85%–90% confluency.

#### 2.1.3 Animals

Forty specific pathogen-free male C57BL/6 mice (18–22 g) were purchased from Liaoning Changsheng Biotechnology Co., Ltd., with the certificate number SCXK (Liaoning) 2020-0001. All animal experiments strictly followed the Guidelines for the Care and Use of Laboratory Animals (NIH Publications No. 85-23, revised 1996) and the Chinese national standard GB/T 35,892-2018, Experimental Animal Welfare and Ethics Review Guide. The 3R principles (Replacement, Reduction, and Refinement) and the Five Freedoms of animal welfare were rigorously adhered to. The experimental protocol was reviewed and approved by the Institutional Animal Welfare and Ethics Committee of Shenyang Pharmaceutical University (Permit Number: SYK (Liaoning) 2021-0009). The animal housing environment was maintained according to GB 14925–2023 standards, including temperature (20 °C–23 °C), relative humidity (50%–65%), and a 12-h light/dark cycle. Prior to experimentation, the animals underwent a 7-day acclimatization period.

### 2.2 Preparation of AVp-EVMs

Fresh whole *Aloe vera* leaves (Haikou, Hainan, China) were thoroughly washed and soaked for 3–5 days until the deionized water became clear and colorless to remove aloin (a bioactive anthraquinone compound found in *Aloe vera*). The leaf spines were excised, and the peel was separated from the gel. Residual gel adhering to the peel was carefully scraped off using a metal spatula. The peel was cut into smaller pieces, mixed with phosphate-buffered saline (PBS) at a mass ratio of 3:1 (peel: PBS), and juiced using a blender. The resulting juice was filtered through nylon mesh and centrifuged. Sequential differential centrifugation steps were performed at 3,000 × g for 30 min and 10,000 × g for 1 h. These steps removed cellular debris, pectin, and other impurities, thereby preventing clogging of the high-pressure homogenizer and ensuring its continuous and stable operation. The resulting supernatant was collected and processed by high-pressure homogenization (Ultra-high Pressure Homogenizer SCIENTZ-207B; Ningbo Scientz Biotechnology Co., Ltd., Ningbo, China) at a pressure of 100 MPa for 10 cycles. This specific parameter set was selected based on an established protocol for preparing functional vesicles from plant material (Abraham et al., 2022) and further optimized in our pilot studies to achieve effective tissue disruption while preserving vesicle integrity and bioactivity. The homogenized liquid was sequentially filtered through 0.45 μm and 0.22 μm microporous membranes. The filtrate was then ultracentrifuged at 100,000 × g for 30 min (Hitachi, fixed rotor, Tokyo, Japan). Finally, the pellet containing AVp-EVMs was resuspended in filtered PBS and stored at −80 °C until further use.

### 2.3 Characterization of AVp-EVMs

AVp-EVMs were diluted in PBS for analysis of particle size distribution and zeta potential using a Malvern Zetasizer Nano ZS (Malvern Panalytical, United Kingdom). Particle concentration and size distribution were further assessed using a NanoSight LM10 system (Malvern Instruments Ltd.). Samples were diluted 1:100 in PBS, injected via an automated syringe pump, and measured in triplicate (30 s/run, 30 frames/second). Particle concentrations were normalized to the 30–150 nm size range. Vesicular structure was confirmed by transmission electron microscopy (TEM; Hitachi HT7800, Tokyo, Japan) following negative staining with 2% (*w/w*) uranyl acetate for 5 min on carbon-coated copper grids. Protein content in AVp-EVMs was quantified by the bicinchoninic acid assay (BCA assay, Beyotime Biotechnology, P0012, Shanghai, China). A 200 µL aliquot of BCA working reagent was prepared according to the manufacturer’s protocol. Subsequently, 25 µL of sample or PBS (blank) was added to a microplate, mixed with 200 µL working solution, and incubated at 37 °C for 30 min before determining the absorbance at 562 nm. Protein concentration (μg/μL) was determined using a standard curve. The molecular weight distribution of proteins was analyzed by Sodium dodecyl sulfate - polyacrylamide gel electrophoresis (SDS-PAGE).

### 2.4 Intervention of AVp-EVMs on HaCaT cells

#### 2.4.1 Apoptosis analysis by flow cytometry

Apoptosis was evaluated using an Annexin V-FITC/PI apoptosis detection kit per the manufacturer’s instructions. HaCaT cells (8 × 10^5^ cells/well) in logarithmic growth were seeded into six-well plates and cultured overnight. After treatment with AVp-EVMs (50, 500, or 700 μg/mL) for 24 h, the supernatant was collected. Cells were washed twice with 1 mL culture medium, and the washes were pooled with the supernatant. Cells were detached using 400 μL trypsin without ethylenediaminetetraacetic acid (EDTA) for 2 min, and the reaction was stopped with 1 mL medium. After centrifugation at 800 rpm for 5 min, the pellet was washed twice with PBS and resuspended in 200 μL PBS. Cell concentration was adjusted to 5 × 10^5^ cells/mL, and the suspension was centrifuged again. Subsequent staining and flow cytometric analysis were performed as described in the kit protocol (BD Biosciences, US).

#### 2.4.2 Cell proliferation assay

HaCaT cell viability was assessed using a cell counting kit-8 assay kit (CCK-8 assay kit, Beyotime Biotechnology, C0037, Shanghai, China). HaCaT cell suspensions (2 × 10^4^ cells/mL) were seeded into 96-well plates at 100 μL/well and treated with AVp-EVMs at concentrations ranging from 50 to 700 μg/mL (200 μL/well, n = 3) for 24 or 48 h. Subsequently, 10 μL CCK-8 reagent was added to each well, followed by incubation at 37 °C for 2 h. Absorbance was determined at 450 nm. All experiments were performed in triplicate. Cell viability (%) was calculated using the formula: Viability (%) = (OD_test_/OD_control_) × 100.

#### 2.4.3 Observation for endocytosis effect

Briefly, phagocytic uptake of PKH-26-labeled AVp-EVMs by HaCaT cells was examined. AVp-EVMs containing 10–200 μg exosomal protein were mixed with 50 μL PKH-26 dye (Umibio Co.Ltd., Shanghai, China), vortexed for 1 min, and incubated at room temperature for 10 min. After adding 7 mL PBS, the labeled EVMs were ultracentrifuged (100,000 rpm, 60 min, 4 °C). The pellets were resuspended in 200 μL PBS, filtered through a 0.22 μm membrane, and quantified by BCA assay. HaCaT cells (1.5 × 10^5^ cells/well) were seeded on coverslips in 24-well plates. After 24 h, PKH-26-labeled AVp-EVMs were added and incubated for 0, 0.5, 4, and 8 h. The cells were washed three times with PBS, fixed with 4% paraformaldehyde (PFA) for 20 min, stained with 4′,6-Diamidino-2′-phenylindole (DAPI, 300 μL/well, 15 min, dark), and mounted for confocal microscopy.

#### 2.4.4 Wound healing assay

The effect of AVp-EVMs (50 and 700 μg/mL) on HaCaT cell migration was assessed by a wound healing assay. Confluent HaCaT monolayers in six-well plates were scratched with a sterile 200 μL pipette tip. After washing with PBS, cells were maintained in serum-free medium. Wound closure was monitored at 0, 6, 12, and 24 h using an inverted microscope. The percentage of wound closure was quantified using ImageJ software. All experiments were performed in triplicate. The migration rate (%) was calculated as Equation 1:

$$\text{Migration rate }\left(\%\right)=\left[\left(A_{0}\;–\;A_{24}\right)/A_{0}\right]\times 100\;$$ (1)

Average wound area at 0 h and 24 h

#### 2.4.5 Transwell assay

HaCaT cell migration was further evaluated by a transwell assay with AVp-EVMs at 50, 500, and 700 μg/mL. HaCaT cells (5 × 10^4^ cells/mL in serum-free DMEM) were seeded into the upper chambers (100 μL per chamber). The lower chambers contained 500 μL complete growth medium. After 24 h of incubation at 37 °C under 5% CO_2_, non-migrated cells on the upper surface were removed with a cotton swab. Migrated cells were fixed with 4% PFA for 10 min, stained with 0.1% crystal violet for 15 min, and counted under an inverted microscope using ImageJ. All experiments were performed in triplicate.

### 2.5 Anti-inflammatory effects of AVp-EVMs

The effect of AVp-EVMs on RAW264.7 cell viability was initially evaluated using a CCK-8 assay kit (Beyotime Biotechnology, C0037). The anti-inflammatory potential of AVp-EVMs was subsequently assessed in LPS-stimulated RAW264.7 cells by quantifying pro-inflammatory cytokine secretion. Cells (2 × 10^5^ cells/well) were pretreated with AVp-EVMs at concentrations of 50, 100, 500, or 700 μg/mL for 12 h, followed by stimulation with LPS (1 μg/mL) for another 12 h. Nitric oxide (NO) levels in the supernatant were determined using Griess reagent (Beyotime Biotechnology, S0021) according to the manufacturer’s instructions. TNF-α, IL-1β, and IL-6 concentrations were determined using ELISA kits (LiankeBio, Hangzhou, China; TNF-α: EK0527, IL-1β: EK0393, IL-6: EK0411) following the provided protocols. Absorbance was determined at 450/630 nm.

### 2.6 Real-time quantitative PCR experiment

Total RNA was extracted from RAW264.7 cells using TRIzol® reagent (Invitrogen, Thermo Fisher Scientific, Waltham, MA, United States) and reverse-transcribed into cDNA. The expression levels of inflammation-related genes (iNOS, COX-2, IL-1β, IL-6, and TNF-α) were quantified by SYBR Green-based qPCR. Primer sequences are provided in Supplementary Table S1. All experiments were conducted in triplicate.

### 2.7 Western blot analysis

Total protein was extracted from RAW264.7 cells using protein extraction buffer, and the protein concentration was determined with a BCA assay kit. Proteins were separated by electrophoresis on 4%–15% precast gels (MeilunBio, Dalian, China) and transferred onto PolyVinylideneFluoride (PVDF) membranes (Merck Millipore, Billerica, MA, United States). GAPDH served as the internal reference. Primary antibodies included inducible nitric oxide synthase (iNOS, T55993S, Abbimart, Shanghai, China; 1:2,000), Cyclooxygenase-2 (COX-2, T58852S, Abbimart, 1:2,000), and Nuclear factor kappa-B (NF-κB, TA5006S, Abbimart, 1:2,000). The secondary antibody was horseradish peroxidase-labeled Goat Anti-Rabbit IgG (HRP-IgG, KGI Biotechnology, Shanghai, China; 1:5,000). Protein bands were visualized using SuperSignal™ West Pico ECL substrate (Pierce Biotechnology, Rockford, IL, United States) and imaged on a ChemiDoc MP system (Bio-Rad, Hercules, CA, United States). All experiments were performed in triplicate.

### 2.8 Preparation of hydrogels

#### 2.8.1 Preparation of non-ionic hydrogel

Sangelose 60L powder (Hydroxypropyl Methylcellulose Stearoxy Ether, Daido chemical corporation., Osaka, Japan) was dispersed in the aqueous solution containing AVp-EVMs, and continued stirring to obtain a uniform Sangelose hydrogel loaded with AVp-EVMs (Sangelose 60L, 0.8%, *w/w*; AVp-EVMs, 4 mg/mL). It was stored at 4 °C for further use.

#### 2.8.2 Preparation of ionic hydrogel

Carbomer 940 powder (Macklin Biochemical Co., Ltd., China) was dispersed in the aqueous solution containing AVp-EVMs. Triethanolamine (Shandong Yousuo Chemical Technology Co., Ltd.) was added to adjust the pH, and was continued stirring to obtain a uniform Carbomer hydrogel loaded with AVp-EVMs with a pH of around 6.5 (Carbomer 940, 0.8%, *w/w*; AVp-EVMs, 4 mg/mL). It was stored at 4 °C for further use.

### 2.9 Hydrogel rheological behavior characterization

MCR-101 rheometer (Anton Paar GmbH, Graz, Austria) was used to determine the storage modulus (G′) and loss modulus (G″) (0.01% strain). The measurements were performed at 25 °C and in the scanning range of 0.1–10 Hz.

### 2.10 Scanning electron microscope

Sangelose 60L and Carbomer 940 hydrogels, both loaded with AVp-EVMs, which had been freeze-dried and cut to size, were affixed to a conductive-glue-coated holder and treated with gold spray. The samples were then observed using a SEM (Hitachi High-Technologies Corp., Tokyo, Japan).

### 2.11 *In vitro* sustained release behavior study

The *in vitro* release profile of hydrogels loaded with AVp-EVMs was evaluated by a dialysis bag method and using a BCA protein assay kit (Beyotime) (Hu et al., 2021). Briefly, the hydrogel loaded with AVp-EVMs was immersed in PBS and placed in a dialysis bag (molecular weight range: 8,000–14,000 Da, Beijing Biotopped Technology Co., Ltd., China), subsequently being incubated at 37 °C and 100 rpm in a shaker (SHA-C, Guohua Electric Co., Ltd., China). The release medium was sampled periodically and replaced with fresh buffer. Absorbance was determined at 595 nm by the BCA assay, and the AVp-EVMs concentration was determined via a standard curve.

### 2.12 Animal model establishment and grouping

#### 2.12.1 Animal model establishment

Male C57BL/6 mice were fed a 45% high-fat diet (HFD, Dechi Biotechnology Co., Ltd., Shanghai, China) for 4 weeks. At the end of the 4-week HFD feeding period, mice were fasted for 12 h. After the fast, diabetes was induced via tail vein injection of streptozotocin (STZ, Merck Life Science KGaA, Darmstadt, Germany; 50 mg/kg in 0.1 M citrate buffer, pH 4.5). Blood glucose was measured after 6 days; successful induction was defined as random glucose >11.1 mmol/L (Wen et al., 2022). Eligible diabetic mice were maintained on a standard diet for 4 weeks to stabilize the diabetic state before wounding. Blood glucose was rechecked before wound induction. Under anesthesia induced by intraperitoneal sodium pentobarbital (Sigma-Aldrich; 1%, 50 mg/kg), full-thickness skin wounds (8 mm diameter, 2 mm depth) were created on the shaved dorsum using a sterile biopsy punch (Hisern Medical Group Co., Ltd., Qingdao, China).

#### 2.12.2 Grouping

Mice were randomly allocated to six groups (n = 6 per group):

- Normal control (NC): Healthy mice that received a four-point peri-wound subcutaneous injection of 200 µL Normal Saline (NS, Thermo Fisher Scientific China Co., Ltd., China).
- Diabetic wound (DW): Diabetic mice that received four-point peri-wound subcutaneous injection of 200 µL NS.
- Blank hydrogel (HG): Diabetic mice that received the topical application of Sangelose 60L hydrogel (Daido chemical corporation) to the wound.
- Low-dose AVp-EVMs injection (EVM-L): Diabetic mice that received four-point peri-wound subcutaneous injection of 200 µL AVp-EVMs (0.4 mg/mL).
- High-dose AVp-EVMs injection (EVM-H): Diabetic mice that received four-point peri-wound subcutaneous injection of 200 µL AVp-EVMs (4 mg/mL).
- Hydrogel loaded with AVp-EVMs (HG-EVM): Diabetic mice that received topical application of Sangelose 60L hydrogel loaded with AVp-EVMs (4 mg/mL) to the wound.

All mice were housed individually with free access to food and water. To monitor the wound healing process, digital images of the wounds were obtained on postoperative days 0, 5, 10, and 15.

### 2.13 Sample collection and processing

Wound tissues were collected, fixed in 10% neutral buffered formalin (Beyotime Biotechnology), and paraffin-embedded. Sections were stained with hematoxylin and eosin (H&E), Masson’s trichrome, and immunohistochemical markers. All procedures were performed in triplicate.

### 2.14 Statistical analysis

All experiments were conducted in triplicate. Data were analyzed using GraphPad Prism 8.0 and expressed as mean ± standard deviation (SD). Normality was tested using the Shapiro-Wilk test. The N values represent independent biological replicates. Statistical significance was set at p < 0.05. Differences between two groups were assessed using Student’s t-test, while one-way analysis of variance (ANOVA) followed by appropriate *post hoc* tests was used for comparisons among three or more groups. Flow cytometry data were processed using FlowJo 10.

## 3 Results

### 3.1 Preparation and characterization of AVp-EVMs

**FIGURE 1:**
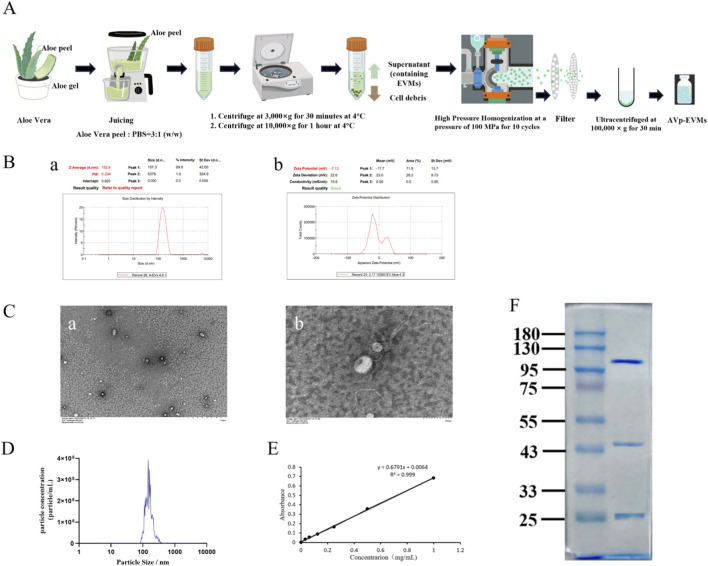
Isolation and characterization of AVp-EVMs. **(A)** Schematic workflow for isolating AVp-EVMs. **(B)** Characterization of AVp-EVMs. a: Particle size distribution, b: zeta potential distribution. **(C)** TEM images of AVp-EVMs. a: ×10,000 magnification, b: ×60,000 magnification. **(D)** NTA-derived size distribution profile of AVp-EVMs. **(E)** Protein quantification standard curve. **(F)** Protein profile of AVp-EVMs by SDS-PAGE.

The procedure for isolating AVp-EVMs is schematically illustrated in Figure 1A. Briefly, following aloin removal, *Aloe vera* peel was juiced and filtered. The resulting filtrate underwent sequential differential centrifugation to remove cellular debris and impurities. The collected supernatant was subjected to high-pressure homogenization. The homogenate was then filtered through microporous membranes, and the filtrate was ultracentrifuged to obtain a concentrated stock solution of AVp-EVMs. The AVp-EVMs stock solution was characterized for size, morphology, particle concentration, and protein concentration. The mean hydrodynamic diameter of AVp-EVMs was determined by dynamic light scattering to be 152.9 nm, with a zeta potential of −7.13 mV (Figure 1B). This relatively low absolute value is commonly associated with limited colloidal stability in dispersion. TEM revealed that AVp-EVMs exhibited a spherical, vesicular morphology (Figure 1C). Nanoparticle tracking analysis (NTA) gave a mean particle size of 175.3 nm and a total particle concentration of 1.8 × 10^10^ particles/mL (Figure 1D). The protein content was determined to be 4.317 mg/mL (Figure 1E), resulting in a particle-to-protein ratio of 4.17 × 10^9^ particles/mg. This preparation served as the standard for all subsequent experiments. Analysis by 10% SDS-PAGE revealed that AVp-EVMs contain multiple proteins, with molecular weights predominantly distributed around 25 kDa, between 43 and 55 kDa, and within the 95–130 kDa range (Figure 1F).

### 3.2 AVp-EVMs promote anti-apoptosis, proliferation, migration, and uptake in HaCaT cells

**FIGURE 2:**
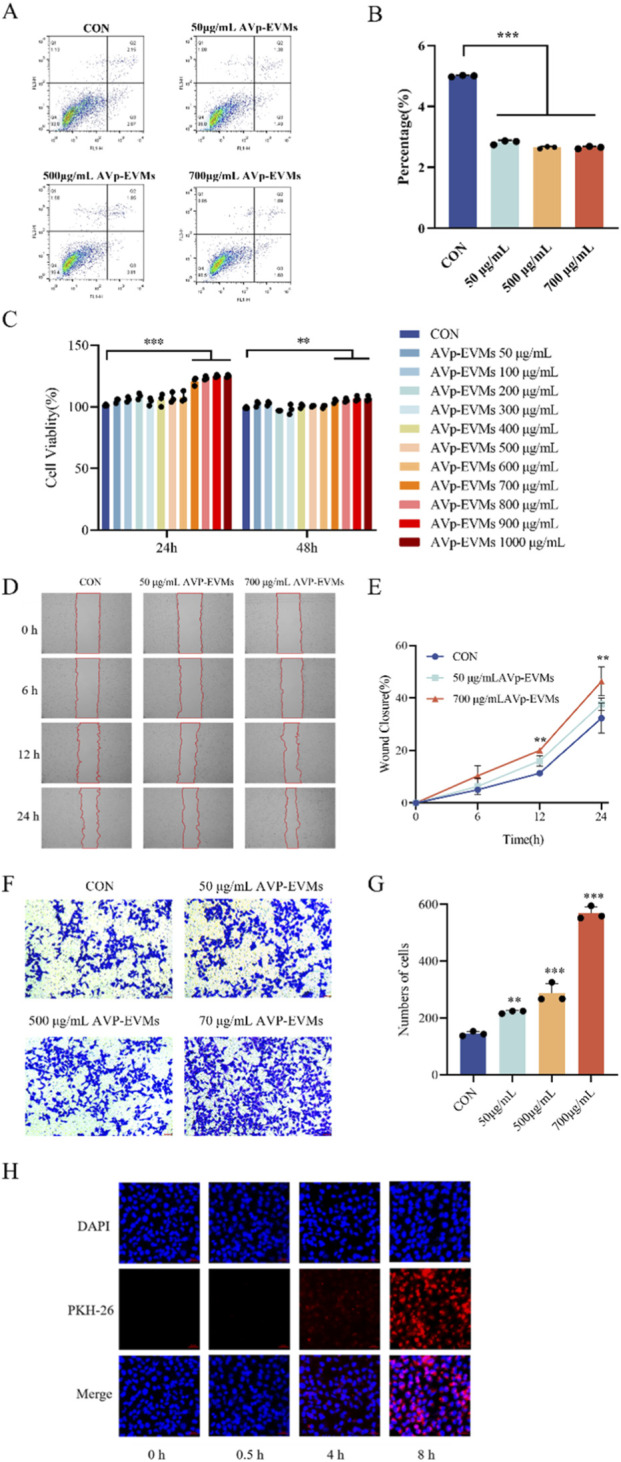
AVp-EVMs promote anti-apoptosis, proliferation, migration, and uptake in HaCaT cells. **(A,B)** Effect of AVp-EVMs on HaCaT cell anti-apoptosis. **(C)** Effect of AVp-EVMs on HaCaT cell proliferation. (D,E) Scratch wound healing assay demonstrating the effect of AVp-EVMs on HaCaT cell migration. **(F,G)** Transwell migration assay (with Matrigel coating) demonstrating the effect of AVp-EVMs on HaCaT cell migration. **(H)** AVp-EVMs uptake by HaCaT cells. Scale bars: 50 μm (representative images). Data are displayed as mean ± SD, n = 3. *P <0.05, **P < 0.01, ***P < 0.001.

Keratinocyte migration over the wound bed is essential for effective wound healing during the re-epithelialization phase. To evaluate the anti-apoptotic capacity of AVp-EVMs, the effect of different AVp-EVMs concentrations on HaCaT cell apoptosis was assessed by Annexin V-FITC/PI double staining combined with flow cytometry. As shown in Figures 2A,B, treatment with AVp-EVMs (50, 500, and 700 μg/mL) for 24 h significantly reduced the apoptotic rate compared to the blank control (*P* < 0.001). Cell proliferation assays (Figure 2C) further demonstrated that within the concentration range of 50–600 μg/mL, cell viability remained >90% after both 24 and 48 h of treatment, showing no significant difference from the control group. This confirmed the absence of cytotoxic effects of AVp-EVMs on HaCaT cells. A significant enhancement in proliferation was observed at 700 μg/mL after 24 h (*P* < 0.001), and this elevated viability was sustained after 48 h (*P* < 0.01). Although a dose-dependent increase in proliferation was noted, concentrations beyond 700 μg/mL did not yield a further statistically significant enhancement. Collectively, these results indicated that 700 μg/mL represented the optimal concentration for eliciting a maximal proliferative response without evidence of cytotoxicity, justifying its selection as the upper dose for follow-up experiments. Building upon these beneficial effects, the regulatory impact of AVp-EVMs on HaCaT cell migration was investigated. Scratch wound assays indicated that intervention with 700 μg/mL AVp-EVMs for 12 h significantly promoted HaCaT cell migration and accelerated wound closure (*P* < 0.01, Figures 2D,E). Transwell migration assays, which specifically assess single-cell chemotactic motility, corroborated the distinct regulatory advantage of AVp-EVMs on keratinocyte migratory behavior (Figures 2F,G). To evaluate the cellular uptake capacity of HaCaT cells for AVp-EVMs, the cells were incubated with PKH-26 fluorescently labeled AVp-EVMs at 37 °C for 0.5, 4, and 8 h. As shown in Figure 2H, a significant increase in red fluorescence intensity was observed at 8 h, indicating the active internalization of AVp-EVMs by HaCaT cells.

### 3.3 AVp-EVMs attenuate LPS-induced inflammatory response in RAW264.7 cells

**FIGURE 3:**
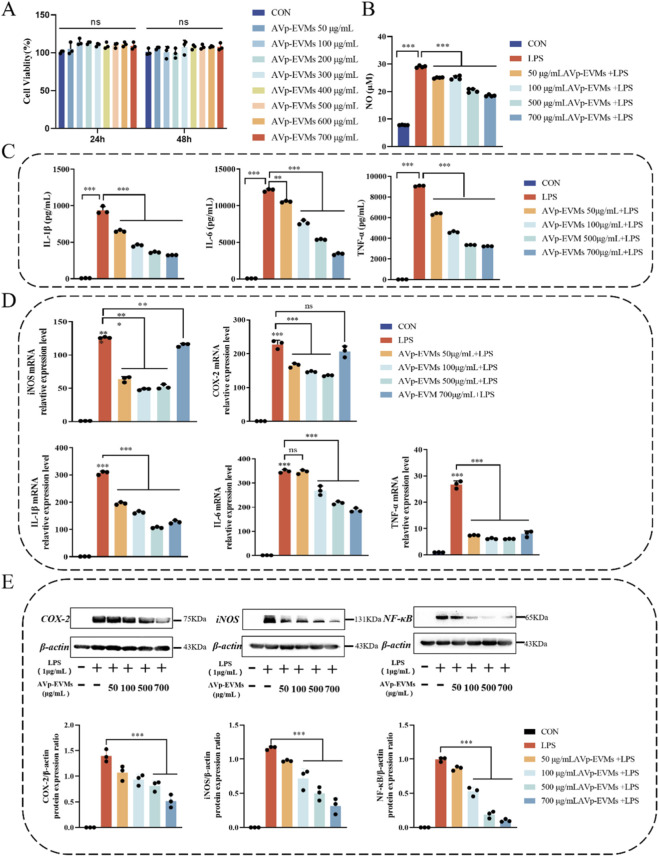
AVp-EVMs attenuate LPS-induced inflammatory responses in RAW264.7 cells. **(A)** Effect of AVp-EVMs on RAW264.7 cell viability. **(B)** Effect of AVp-EVMs on LPS-induced nitric oxide (NO) production in RAW264.7 cells. **(C)** Effect of AVp-EVMs on LPS-induced secretion of pro-inflammatory cytokines in RAW264.7 cells. **(D)** Effect of various AVp-EVMs concentrations on the mRNA expression levels of pro-inflammatory cytokines in LPS-stimulated RAW264.7 cells (qRT-PCR analysis). **(E)** Effect of various AVp-EVMs concentrations on the protein expression levels of pro-inflammatory cytokines in LPS-stimulated RAW264.7 cells (immunoblotting analysis). Data are displayed as mean ± SD, n = 3. *P < 0.05, **P < 0.01, ***P < 0.001.

To evaluate the regulatory role of AVp-EVMs on macrophage inflammatory response, we first confirmed through cell viability assays that after 24 or 48 h of treatment within the concentration range of 50–700 μg/mL, AVp-EVMs maintained >90% viability of RAW264.7 cells compared with the control group, indicating no cytotoxicity (Figure 3A). Compared with the control, NO levels were reduced in a dose-dependent manner in all treatment groups, with the most significant reduction observed at 700 μg/mL AVp-EVMs (Figure 3B). IL-1β, IL-6, and TNF-α, as key inflammatory mediators, play central roles in the regulation of inflammatory and immune responses. Therefore, the IL-1β, IL-6, and TNF-α levels in the culture supernatant were determined using ELISA kits. The results showed that, compared with the CON group, LPS stimulation significantly increased the IL-1β, IL-6, and TNF-α levels in the supernatant (*P* < 0.001), while all treatment groups effectively suppressed the release of these key pro-inflammatory cytokines (Figure 3C). qPCR results demonstrated that AVp-EVMs significantly downregulated iNOS, COX-2, IL-1β, IL-6, and TNF-α mRNA expression (Figure 3D). Western blot analysis indicated that the elevated expression of key proteins, including COX-2, iNOS, and NF-κB, in LPS-induced RAW264.7 macrophages was dose-dependently inhibited by AVp-EVMs, with the most pronounced inhibition observed at 700 μg/mL (*P* < 0.01, Figure 3E).

### 3.4 Preparation and characterization of HG-EVM

**FIGURE 4:**
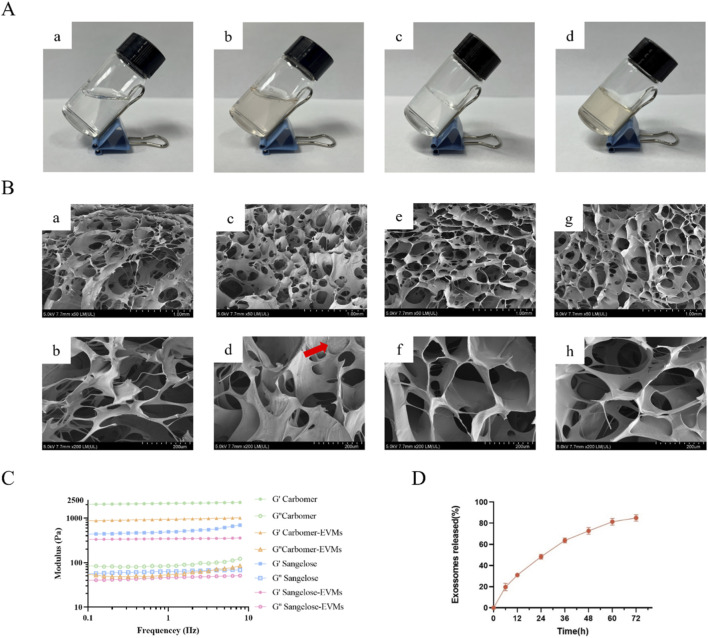
Characterization of HG-EVM. **(A)** Macroscopic morphology of hydrogels: (a) Blank Sangelose 60L hydrogel, (b) Sangelose 60L hydrogel loaded with AVp-EVMs, (c) blank Carbomer 940 hydrogel, (d) Carbomer 940 hydrogel loaded with AVp-EVMs. **(B)** SEM images of all hydrogels. Blank Sangelose 60L hydrogel: (a) ×50 magnification, (b) ×200 magnification; Sangelose 60L hydrogel loaded with AVp-EVMs: (c) ×50 magnification, (d) ×200 magnification; blank Carbomer 940 hydrogel: (e) ×50 magnification, (f) ×200 magnification; Carbomer 940 hydrogel loaded with AVp-EVMs: (g) ×50 magnification, (h) ×200 magnification. Scale bars: 1 mm (a, c, e, g), 200 μm (b, d, f, h). **(C)** Rheological analysis of hydrogels under frequency sweep (storage modulus G′ and loss modulus G″). **(D)** Cumulative release profile of AVp-EVMs from Sangelose 60L hydrogel *in vitro*. Data are displayed as mean ± SD, n = 3.

Sangelose 60L and Carbomer 940 hydrogels loaded with AVp-EVMs were prepared for wound healing therapy. As shown in Figure 4A, the blank Sangelose 60L hydrogel (Figure 4A-a) appeared transparent and exhibited slight flowability without wall adhesion when tilted at 45°. By contrast, the blank Carbomer 940 hydrogel (Figures 4A-c) demonstrated high viscosity and remained static upon tilting. Both Sangelose 60L hydrogel loaded with AVp-EVMs (Figures 4A-b) and Carbomer 940 system loaded with AVp-EVMs (Figures 4A-d) exhibited a pale yellow color while maintaining their respective flow properties. No particle sedimentation was observed, indicating homogeneous dispersion and good compatibility between AVp-EVMs and both hydrogel matrices. Neither loaded hydrogel displayed phase separation or flocculation after 72 h of static storage, confirming their colloidal stability. SEM revealed that freeze-dried samples of both hydrogels displayed characteristic three-dimensional interconnected porous networks (Figure 4B). High-magnification imaging identified distinct spherical nanoparticles, corresponding to nanovesicles, on the pore walls of the Sangelose 60L-EVMs hydrogel (Figures 4B-d). Conversely, the Carbomer 940-EVMs hydrogel displayed a complete absence of intact vesicular structures (Figures 4B-h), indicating stable preservation of AVp-EVMs exclusively in the Sangelose 60L system. Rheological analysis (Figure 4C) demonstrated that across the 0.1–10 Hz frequency range, the storage modulus (G′) consistently predominated over the loss modulus (G″) for all formulations, characteristic of elastic-dominant materials. Given the instability of AVp-EVMs in the Carbomer system, *in vitro* release testing was performed only for the Sangelose 60L-EVMs hydrogel. Release studies (Figure 4D) showed a 74% cumulative release of AVp-EVMs at 48 h, with sustained release continuing through 72 h. These results demonstrated the potential of Sangelose 60L hydrogel as a sustained-release platform for prolonging vesicle retention at target sites.

### 3.5 AVp-EVMs and HG-EVM accelerate diabetic wound healing in mice

**FIGURE 5:**
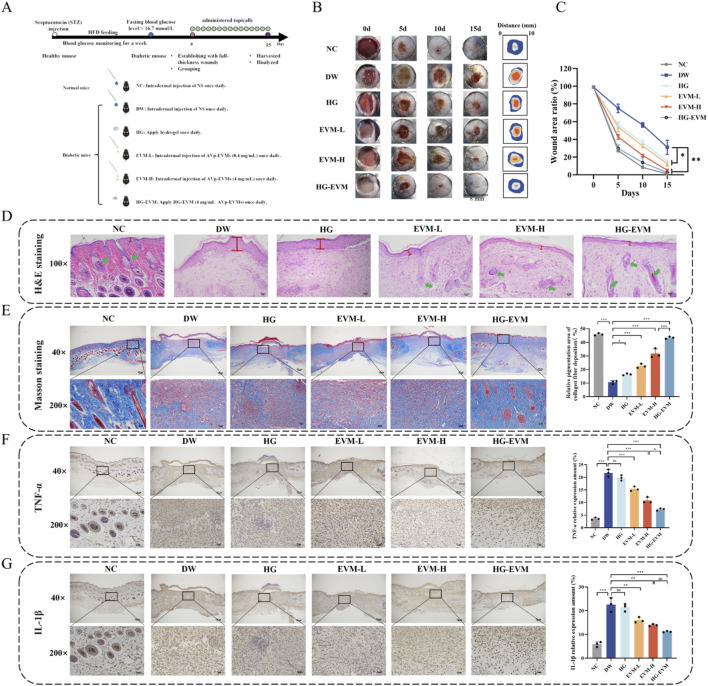
HG-EVM promotes wound healing in diabetic mice. **(A)** Schematic diagram of the mouse experimental design. **(B)** Representative images of dorsal wounds from each group (Days 0, 5, 10, 15 post-wounding). **(C)** Quantification of the wound area ratio (%). **(D)** H&E staining and representative images of wound tissue sections (The red line represents epidermal thickness, and the green arrow represents the skin appendages). **(E)** Masson’s trichrome staining (representative images) and quantitative analysis of collagen content in wound tissues. **(F)** Immunohistochemical (IHC) analysis and quantification of TNF-α expression in wound tissues (representative images). **(G)** IHC analysis and quantification of IL-1β expression in wound tissues (representative images). Data are displayed as mean ± SD, n = 6. *P < 0.05, **P < 0.01, ***P < 0.001.

To validate the effectiveness of HG-EVM in healing diabetic wounds, a full-thickness skin wound was created in mice. The experimental dosing regimen and group allocation are illustrated in Figure 5A. As illustrated in Figures 5B,C, after 15 weeks of treatment, the NC group exhibited complete closure. The wounds in the DW group (69%) did not heal completely. This revealed an impaired healing process. The significantly increased healing rates observed in the EVM-L (87.67%) and EVM-H (94.83%) groups demonstrated the capability of AVp-EVMs to promote wound repair. Notably, a negligible wound size was observed in the HG-EVM group (97.16%). This indicated that HG-EVM was highly effective in improving the healing of the diabetic wound. To further evaluate the healing-promoting capacity of HG-EVM, wound tissue sections were analyzed at day 15 post-treatment by H&E staining, Masson’s trichrome staining, and immunohistochemistry (IHC). Histopathologic examination (Figure 5D) revealed that compared to the NC group, the DW group exhibited a markedly thickened epidermis with granular layer hyperplasia, together with densely packed and disorganized collagen fiber bundles. Although the HG group displayed a smoother epidermal layer, the epidermal thickness exceeded that of the EVM-L group. Both the EVM-H and HG-EVM groups exhibited a relatively thinner epidermis with a well-defined structure, looser collagen fibers, and a more regular arrangement. Notably, nascent hair follicles were clearly observed in the HG-EVM group. Masson’s trichrome quantification (Figure 5E) demonstrated the lowest collagen deposition in the DW group versus the NC group. While the HG group exhibited higher collagen deposition than the DW group, it remained significantly lower than the EVM-L and EVM-H groups. The HG-EVM group achieved the highest collagen deposition with optimal alignment of newly formed collagen fibers, indicating enhanced extracellular matrix reconstruction and tissue remodeling. IHC analysis of inflammation-related biomarkers TNF-α and IL-1β (Figures 5F,G) showed increased expression of both cytokines in the DW group compared with the NC group. HG treatment did not reduce TNF-α or IL-1β expression, and no significant difference in expression levels between the HG and DW groups. By contrast, both the EVM-L and EVM-H groups significantly reduced TNF-α and IL-1β expression compared to the DW group (*P* < 0.01), confirming the anti-inflammatory properties of AVp-EVMs. Compared with the EVM-H group, the HG-EVM group demonstrated enhanced suppression of TNF-α (*P* < 0.05) and a trend toward greater reduction in IL-1β expression.

## 4 Discussion

We developed a production method with good potential for scalability for producing AVp-EVMs by coupling high-pressure homogenization with ultracentrifugation. The high-pressure homogenization step serves as an efficient pretreatment that disrupts plant cell walls to fully release intracellular vesicles, which are then mixed and recombined with pre-existing extracellular vesicle aggregates. This process significantly enhances the efficiency and scalability of subsequent ultracentrifugation. This integrated approach effectively resolved the AVp-EVMs production bottlenecks. In contrast to the typical “saucer-like” morphology of natural exosomes (Thery et al., 2018), TEM revealed that AVp-EVMs exhibited homogeneous spherical nanostructures. This morphological reshaping is attributed to the intense fluid shear forces during homogenization coupled with physical restructuring via membrane filtration. We hypothesize that a uniform size distribution may enhance cellular endocytic efficiency (Rejman et al., 2004), providing a foundation for future research.

During wound healing, apoptotic cell accumulation in wound microenvironments is one of the key factors impeding re-epithelialization. Our findings demonstrated that AVp-EVMs significantly suppress spontaneous apoptosis in HaCaT keratinocytes, indicating their capacity to maintain cellular homeostasis. This effect likely sustains the cellular reservoir essential for re-epithelialization, indirectly promoting wound healing (Wilgus et al., 2013). Furthermore, we observed that AVp-EVMs accelerated HaCaT migration, a critical determinant of successful re-epithelialization (Pastar et al., 2014). Confocal microscopy confirmed that AVp-EVMs were efficiently internalized by HaCaT cells in a time-dependent manner. This observation suggests that AVp-EVMs are actively internalized by cells, a process that may involve multiple pathways such as endocytosis (including pinocytosis) and membrane fusion, consistent with findings reported for aloe-derived extracellular vesicles (Zeng et al., 2022). More importantly, these results directly demonstrate that the preparation method employed—high-pressure homogenization combined with ultracentrifugation—successfully yielded vesicles with intact structure and well-preserved bioactivity, whose functionality was not compromised by the high-pressure homogenization process. This provides a solid foundation for subsequent investigations into their biological functions and potential applications.

Regarding inflammation modulation, a moderate inflammatory response is necessary during the early stages of wound healing, whereas excessive or prolonged inflammation can delay the healing process (Eming et al., 2007). A key pathologic feature of the diabetic wound microenvironment is aberrant NF-κB activation. This abnormal activation not only induces elevated pro-inflammatory cytokine levels (TNF-α, IL-1, IL-6, Cluster of differentiation 36, Monocyte chemoattractant protein-1) that promote inflammation and lead to endothelial cell death. (Evans and Goldfine, 2016), but also upregulates COX-2 and iNOS expression, further exacerbating inflammation (Kim et al., 2000; Tan et al., 2019). Our study demonstrated that AVp-EVMs effectively suppress NF-κB pathway activation. In an LPS-stimulated RAW264.7 macrophage model, AVp-EVMs significantly inhibited iNOS, COX-2, IL-1β, IL-6, and TNF-α mRNA expression. They also downregulated COX-2, iNOS, and NF-κB protein expression while reducing pro-inflammatory cytokine secretion (IL-1β, IL-6, TNF-α). These results collectively confirmed the pronounced anti-inflammatory activity of AVp-EVMs (Koh and DiPietro, 2011; Krzyszczyk et al., 2018). By attenuating NF-κB-mediated excessive inflammation, AVp-EVMs ameliorate the wound microenvironment, thereby creating favorable conditions for re-epithelialization (Landén et al., 2016). While our data demonstrate suppression of total NF-κB expression and its downstream inflammatory mediators, future studies employing techniques such as immunofluorescence to track p65 nuclear translocation will be valuable to further delineate the precise step at which AVp-EVMs inhibit this pathway.

To address delivery challenges of free vesicles, such as dilution by tissue fluid and immune clearance (Chabria et al., 2021; Yan et al., 2023), we employed hydrogels as AVp-EVMs carriers. During the screening of matrix materials, we focused on comparing two commonly used hydrogels: Sangelose®, a novel hydrophobically modified hydroxypropyl methylcellulose derivative, and carbomer, an allyl sucrose-crosslinked polyacrylic acid anionic polymer. During the screening process, it was observed that the carbomer hydrogel system exhibited higher viscosity than the Sangelose 60L hydrogel system. This difference primarily stems from the distinct molecular characteristics of the two hydrogels. As an ionic polymer, Carbomer 940 ionizes in solution and generates strong electrostatic repulsion, thereby increasing the viscosity of the system. By contrast, Sangelose 60L, due to its non-ionic hydrophobic association properties, forms a hydrogel system with relatively lower viscosity (Wang et al., 1988). Further observation through SEM revealed that the nanovesicles exhibit greater stability in non-ionic hydrogel systems compared to ionic ones. The core advantage of Sangelose 60L lies in its non-ionic nature, which provides a neutral and mild pH environment crucial for maintaining AVp-EVMs structural integrity. In contrast, ionic hydrogels such as Carbomer possess inherent charges that may induce electrostatic repulsion with charged nanovesicles, potentially compromising vesicle structural stability (Carrión et al., 1994; Wang et al., 2019). Therefore, this study ultimately selected Sangelose 60L as the delivery carrier for AVp-EVMs. This hydrogel, with its three-dimensional network structure, enables sustained AVp-EVMs release over 72 h, effectively overcoming the limitations of subcutaneous injection of free vesicles. Furthermore, the encapsulation within the hydrogel matrix directly addresses the colloidal stability challenge inherent to nanovesicle dispersions. The measured zeta potential of AVp-EVMs suggests a tendency for aggregation in aqueous suspension over time. The Sangelose hydrogel’s network physically entraps and separates individual vesicles, preventing their contact and aggregation through steric stabilization. This transforms the AVp-EVMs from a labile dispersion requiring cryopreservation into a stable, ready-to-use topical formulation.

On the basis of the positive results from *in vitro* findings and the comprehensive advantages of non-ionic hydrogels, we evaluated AVp-EVMs and HG-EVM in a diabetic wound model. The experimental results confirmed that the HG group exerted moderate yet positive effects on epidermal regeneration and collagen deposition, confirming its potential as a hydrogel dressing for wound repair. Subcutaneous AVp-EVMs injection significantly promoted diabetic wound healing, as evidenced by enhanced epidermal repair, increased collagen deposition, and suppression of pro-inflammatory cytokine expression. Critically, the HG-EVM group demonstrated markedly superior efficacy in promoting diabetic wound healing compared to subcutaneous AVp-EVMs injection alone. These results indicate that the therapeutic benefits of HG-EVM are primarily attributable to the bioactivity of AVp-EVMs. The present study was designed to validate the efficacy of this combined formulation and to compare it with free EVM injection; therefore, control groups such as hydrogel loaded with crude *Aloe vera* peel extract were not included. It should also be noted that generating plant-derived vesicles completely devoid of endogenous bioactive molecules remains technically infeasible, and synthetic liposome controls would not adequately replicate the membrane composition and physical properties of native nanovesicles. Importantly, our cellular uptake experiments (Figure 2H) demonstrated that AVp-EVMs are internalized by HaCaT cells in a time-dependent manner, confirming their cellular affinity, a prerequisite for effective intracellular cargo delivery. This observation is consistent with previous reports demonstrating that Aloe vera peel-derived extracellular vesicles are internalized by HaCaT cells via multiple endocytic pathways, including clathrin- and caveolae-mediated endocytosis, as well as membrane fusion (Kim et al., 2021). Furthermore, the advantage of vesicle-mediated delivery over free compounds has been demonstrated in HaCaT cells: astaxanthin-loaded extracellular vesicles (EV-ASTs) exhibited significantly higher antioxidant and anti-inflammatory activities than free astaxanthin at equivalent concentrations, confirming the enhanced delivery efficiency conferred by vesicle encapsulation (Jang et al., 2023). Together with the dose-dependent efficacy of AVp-EVMs and the blank hydrogel control, these data support that the observed *in vivo* effects are attributable to the biological activity of the vesicles themselves rather than to nonspecific matrix effects.

The observed bioactivity of AVp-EVMs is likely rooted in the unique composition of the *Aloe vera* peel. As noted, our comparative *in vitro* data (Supplementary Figure S1) indicated that AVg-EVMs had a weaker proliferative effect, suggesting that specific components enriched in the peel are crucial. *Aloe vera* has a long history of use in traditional medicine, primarily due to bioactive compounds such as acemannan and anthraquinones, which promote wound healing by stimulating cell proliferation and reducing inflammation (Massoud et al., 2022). To investigate the material basis of this effect, we performed high-performance liquid chromatography using aloin as a representative anthraquinone in Aloe vera and detected trace amounts of aloin in concentrated AVp-EVMs stock solution (Supplementary Figure S2). This indicates that although the vast majority of anthraquinones were removed from the Aloe vera peel by soaking, trace amounts of anthraquinones were still encapsulated in AVp-EVMs. Literature reports support the plausibility of our findings: aloin has been shown to accelerate burn healing by prurereover, compared with other parts of Aloe vera (flower, gel, and root), the peel exhibits stronger antioxidant activity (Quispe et al., 2018), which may better alleviate oxidative stress at the wound site. Nevertheless, recent studies have indicated that anthraquinones in *Aloe vera* extracts may cause adverse effects, such as skin irritation (Massoud et al., 2022). This contrast highlights that the trace amount of aloin encapsulated in our prepared AVp-EVMs may fall within a potentially favorable therapeutic window—effective in exerting anti-inflammatory, antioxidant, and wound-healing activities while avoiding potential toxic side effects. Based on the above evidence, we speculate that trace active ingredients such as aloin, along with other bioactive components encapsulated in AVp-EVMs (e.g., nucleic acids and other secondary metabolites from Aloe vera peel), may act synergistically to enhance the positive effects of AVp-EVMs in promoting epidermal cell proliferation and migration, collagen deposition, and reduction of inflammatory responses. It is noteworthy that although acemannan in Aloe vera has been widely reported to possess wound-healing activity (Tabandeh et al., 2014; Xing et al., 2014). As a polysaccharide macromolecule, it is difficult to be effectively encapsulated by nanovesicles; therefore, it is not considered a core active substance in AVp-EVMs. The specific mechanisms underlying the synergistic interactions among these components will be a focus of future research.

On the other hand, this study demonstrates that Sangelose 60L non-ionic hydrogel can effectively maintain the morphological and membrane structural stability of AVp-EVMs. This finding provides critical experimental evidence for selecting suitable hydrogels as delivery matrices for nanovesicles (e.g., EVs, EVMs). Furthermore, the hydrogel delivery system itself contributes to wound healing. For instance, its high water content helps maintain a moist wound environment, reduces transepidermal water loss, and accelerates epithelial cell migration (Ho et al., 2022). The formed physical barrier prevents pathogen invasion, thereby avoiding infection-related healing delays. Additionally, the viscoelastic properties of the hydrogel provide appropriate physical support, promoting cell spreading and migration (Pranantyo et al., 2024). Through integrin-focal adhesion kinase axis activation, it stimulates nuclear translocation of the mechanosensitive transcription factors yes-associated protein/transcriptional coactivator with PDZ-binding motif, upregulating TGF-β and collagen synthesis genes (Brusatin et al., 2018).

This study not only confirms the therapeutic potential of AVp-EVMs in wound healing but also highlights the effectiveness of non-ionic hydrogels as their efficient delivery carriers. However, several limitations remain and warrant further investigation in the future. First, the differences and underlying mechanisms among vesicles from different sources require in-depth analysis. This study found that AVp-EVMs outperformed AVg-EVMs in promoting cell proliferation, suggesting compositional and functional differences between them. Future work will focus on elucidating the mechanisms responsible for these functional disparities. Future work will focus on elucidating the specific membrane proteins and lipid species responsible for the superior cellular affinity of AVp-EVMs using proteomic and lipidomic analyses. Additionally, although AVg-EVMs exhibited limited efficacy in promoting wound healing in this study, their affinity for cell membranes may render them efficient natural intracellular delivery vehicles. It is worthwhile to further investigate their endocytic efficiency and intracellular trafficking pathways, as well as to explore their potential for loading and delivering other therapeutic molecules, such as nucleic acid drugs or small-molecule anti-inflammatory agents. Second, the multi-component synergistic mechanisms of AVp-EVMs need further clarification. Our preliminary data point to trace amounts of aloin as a potential key contributor; unlike nucleic acids, which are often unstable in plant-derived vesicles, small molecule anthraquinones are relatively stable and may synergize with other vesicle components. Future studies should employ multi-omics analyses to comprehensively characterize their component profiles and use fractionation and reconstitution experiments to validate the functional contribution of aloin and other candidate compounds. Finally, to overcome the inherent heterogeneity of native vesicles and to create well-defined controls, we are exploring hybrid vesicle technology (e.g., fusion of natural AVp-EVMs with synthetic liposomes) and bioengineered exosome-mimetic systems. These approaches will enable the generation of customizable, scalable, and stable vesicle-based therapeutics with defined cargo, thereby enabling rigorous mechanism-of-action studies and facilitating clinical translation. Based on the multifunctional properties of AVp-EVMs, future research could explore their combined use with antibacterial therapies, growth factors, or physical treatments to address more complex clinical wound types. Further engineering modifications to enhance their targeting ability may enable more precise and efficient intelligent therapies.

In summary, this study provides new insights into the development of vesicle-based therapies derived from *aloe vera* peels. It not only contributes to elucidating their biological foundations but may also open up new application avenues as natural nano-delivery platforms.

## Data Availability

The raw data supporting the conclusions of this article will be made available by the authors, without undue reservation.

## Glossary

ANOVA: analysis of variance
AVg: Aloe vera gel
AVp: Aloe vera peel
BCA: bicinchoninic acid
CCK8: cell counting kit-8
COX-2: Cyclooxygenase-2
DAPI: 4′,6-Diamidino-2′-phenylindole
DMEM: Dulbecco’s Modified Eagle Medium
EDTA: ethylenediaminetetraacetic acid
EVs: extracellular vesicles
EVPs: extracellular vesicle-like particles
EVMs: extracellular vesicle mimetics
HaCaT: Human immortalized keratinocytes
H&E: hematoxylin and eosin
HCl: Hydrochloric acid
HFD: high-fat diet
HG-EVM: hydrogels loaded with AVp-EVMs
HRP-lgG: horseradish peroxidase-labeled Goat Anti-Rabbit IgG
IHC: immunohistochemistry
IL: nterleukin
iNOS: inducible nitric oxide synthase
LPS: lipopolysaccharide
NF-κB: Nuclear factor kappa-B
NO: Nitric oxide
NS: Normal Saline
NTA: Nanoparticle tracking analysis
PBS: phosphate-buffered saline
PFA: paraformaldehyde
PVDF: PolyVinylideneFluoride
RAW264.7: Murine macrophages
SD: standard deviation
SDS-PAGE: Sodium dodecyl sulfate - polyacrylamide gel electrophoresis
SEM: scanning electron microscope
STZ: streptozotocin
TEM: transmission electron microscopy
TNF: Tumor Necrosis Factor.
