# Correction: Hydroxypropyl methylcellulose stearoxy ether hydrogel loaded with aloe vera peel-derived extracellular vesicle mimetics promotes wound healing in diabetic mice

**DOI:** 10.3389/fbioe.2026.1840527

**Published:** 2026-05-05

**Authors:** Jiatong Wang, Peng Wu, Juan Hu, YiLin Wang, Tianci Lang, Yue Tan, Ge Yu, Shizhu Cai, Ruihan Liu, Yichi Sun, Nan Liu, Fang Wang, Linghe Zang, Dongchun Liu

**Affiliations:** 1 School of Chinese Materia Medica, Shenyang Pharmaceutical University, Shenyang, China; 2 Joint International Research Laboratory of Intelligent Drug Delivery Systems of Ministry of Education, Shen-yang Pharmaceutical University, Shenyang, China; 3 Hainan Enneas Technology Co., Ltd., Haikou, China; 4 School of Pharmacy, Shenyang Pharmaceutical University, Shenyang, China; 5 Faculty of Functional and Wine, Shenyang Pharmaceutical University, Shenyang, China; 6 School of Clinical Pharmacy, Shenyang Pharmaceutical University, Shenyang, China

**Keywords:** aloe vera, diabetic wound healing, extracellular vesicle mimetics, hydrogel delivery system, hydroxypropyl methylcellulose stearoxy ether, sangelose

Author Juan Hu’s affiliation “Hainan Enneas Technology Co., Ltd., Haikou, China” was erroneously given as “Hainan Jiumiantong Technology Co., Ltd., Haikou, China”.

In the published article, there was an error in the text regarding the company name. In both sections, the company name “Daito kasei kogyo Co., Ltd.” has been corrected to “Daido chemical corporation”.

A correction has been made to 2.8 Preparation of hydrogels, 2.8.1 Preparation of non-ionic hydrogel and Section 2.12.2.

“Sangelose 60L powder (Hydroxypropyl Methylcellulose Stearoxy Ether, Daido chemical corporation., Osaka, Japan) was dispersed in the aqueous solution containing AVp-EVMs, and continued stirring to obtain a uniform Sangelose hydrogel loaded with AVp-EVMs (Sangelose 60L, 0.8%, w/w; AVp-EVMs, 4 mg/mL). It was stored at 4 °C for further use.”

A correction has been made to 2.12 Animal model establishment and grouping, 2.12.2 Grouping.

“Blank hydrogel (HG): Diabetic mice that received the topical application of Sangelose 60L hydrogel (Daido chemical corporation) to the wound.”

The original article has been updated.

