# Supplementary material for: Hydroxypropyl methylcellulose stearoxy ether hydrogel loaded with aloe vera peel-derived extracellular vesicle mimetics promotes wound healing in diabetic mice
Source: Front Bioeng Biotechnol. 2026 Mar 25;14:1768930. doi: 10.3389/fbioe.2026.1768930 (PMC13057360; doi:10.3389/fbioe.2026.1768930)
Supplement: Supplementary file 1 [file Supplementaryfile1.docx]

Supplementary Material

# Methods

**Extraction of *Aloe Vera* Gel-Derived Extracellular Vesicle Mimetics (AVg-EVMs)**

Fresh whole *Aloe vera*. L leaves (Haikou, Hainan, China) were thoroughly washed and soaked in deionized water for 3–5 days to remove aloin. The leaf spines were subsequently trimmed off and the peel was separated from the gel. Residual gel adhering to the peel was carefully scraped off using a metal spatula. The gel was directly homogenized using a juice extractor and filtered through nylon mesh to obtain a gel homogenate. Sequential differential centrifugation steps were performed at 3,000 × g for 30 minutes and 10,000 × g for 1 hour. These steps removed cellular debris, pectin, and other impurities, thereby preventing clogging of the high-pressure homogenizer and ensuring its continuous and stable operation. The resulting supernatant was collected and processed by high-pressure homogenization (Ultra-high Pressure Homogenizer SCIENTZ-207B; Ningbo Scientz Biotechnology Co., Ltd., Ningbo, China) at a pressure of 100 MPa for 10 cycles. The homogenized liquid was sequentially filtered through 0.45 μm and 0.22 μm microporous membranes. The filtrate was then ultracentrifuged at 100,000 × g for 30 min (Hitachi, fixed rotor, Tokyo, Japan). Finally, the pellet containing AVg-EVMs was resuspended in filtered PBS and stored at −80°C until further use.

**Screening of AVp-EVMs and AVg-EVMs with HaCaT Human Keratinocytes**

HaCaT cell viability was assessed using a cell counting kit-8 assay kit (CCK-8, Beyotime Biotechnology, C0037, Shanghai, China). HaCaT cell suspensions (2 × 10⁴ cells/mL) were seeded into 96-well plates at 100 μL/well. Cells were subsequently treated with varying AVp-EVMs or AVg-EVMs concentrations (50–700 μg/mL; 200 μL/well, n = 3) for either 24 hours. Following treatment, 10 μL CCK-8 reagent (Beyotime Biotechnology, C0037) was added to each well. After incubation at 37°C for 2 hours, absorbance was determined at 450 nm. Each experiment was performed in triplicate. Cell viability (%) was calculated using the formula: Viability (%) = (OD_test_ / OD_control_) × 100.

**Quantification of Aloin**

Quantitative analysis of aloin was performed by High Performance Liquid Chromatography (HPLC) analysis under optimized chromatographic conditions. Separation employed a Wondaeract ODS-2 column (5 μm, 4.6 × 250 mm, Shimadzu Corporation, Kyoto, Japan) with an isocratic elution of methanol:1% glacial acetic acid (45:55, *v/v*) mobile phase at a 1.0 mL/min flow rate, maintaining column temperature at 40°C. The detection wavelength was set at 359 nm throughout the 20-minute analytical run with 10 μL injection volumes.

# Results

**Supplemental Figure 1**

**
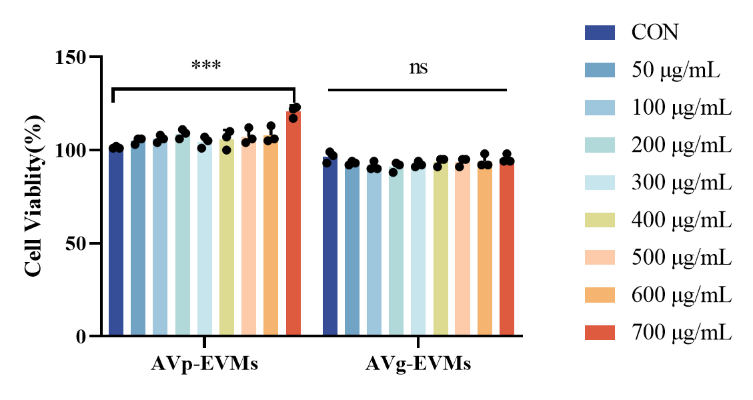
**

**Supplemental Figure 1:** Effects of AVp-EVMs versus AVg-EVMs on HaCaT cell proliferation. Data are displayed as mean ± SD, n = 3. **P* < 0.05, ***P* < 0.01, ****P* < 0.001.

CCK-8 assays revealed that while AVg-EVMs exhibited no significant effect on HaCaT cell proliferation across the tested concentrations, AVp-EVMs (at >700 μg/mL) significantly enhanced proliferation after 24 hours compared to controls.

**Supplemental Figure 2**

**
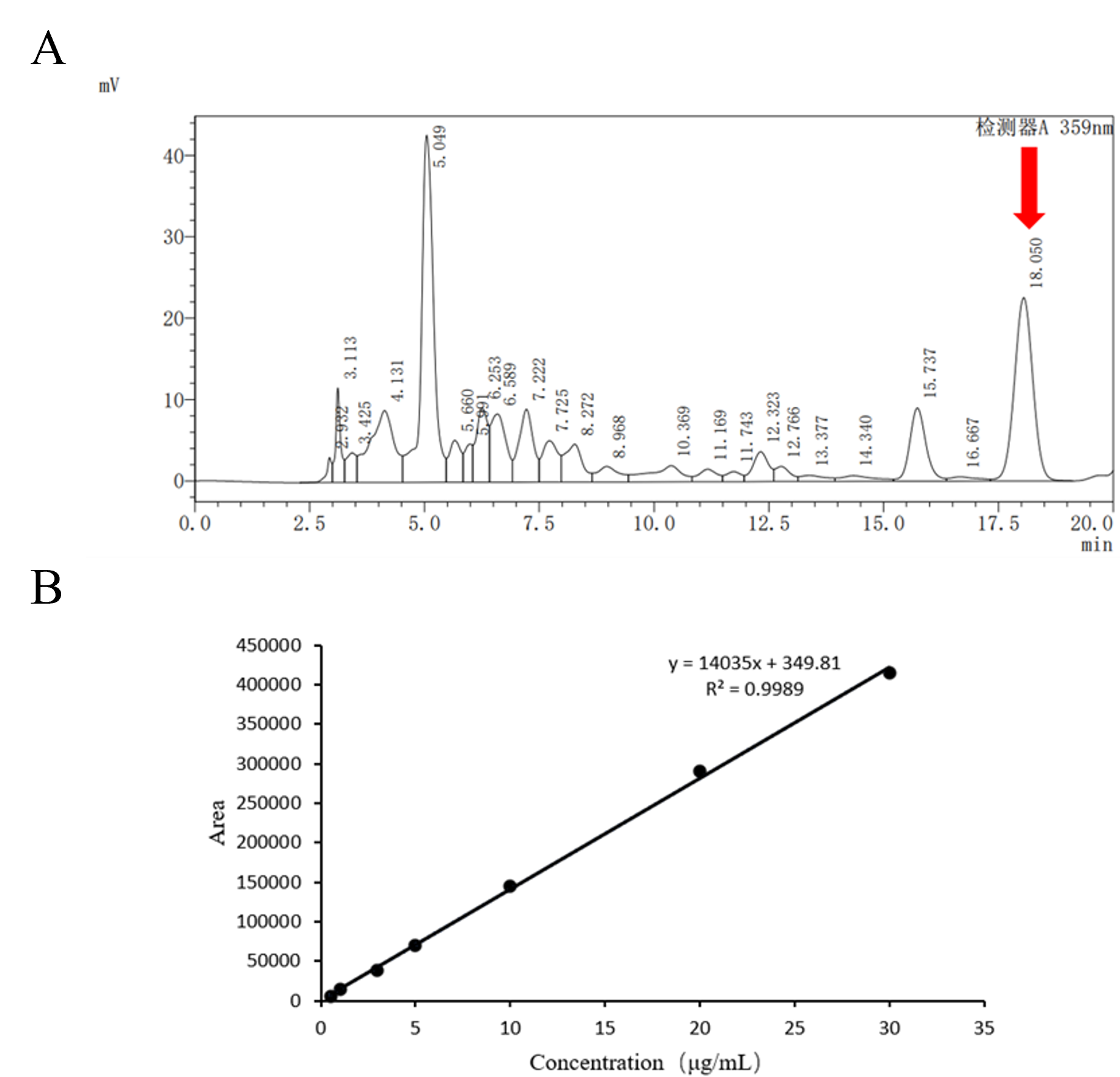
**

**Supplemental Figure 2:** Analysis of AVp-EVMs isolates. (A) HPLC of aloin in extracellular vesicles of AVp-EVMs. (B) Standard curve of aloin.

The aloin reference standard exhibited a retention time of 18.05 min, with the corresponding peak identified in AVp-EVMs chromatograms (indicated by an arrow in Figure S2A). The calibration curve for aloin displayed good linearity within the concentration range of 0.5–30 μg/mL (R² = 0.9994), with a regression equation of Y = 14035.5X + 349.659. The sample aloin concentration was calculated by substituting peak areas into this equation. A concentration of 2.073 μg aloin per mg protein was measured in the AVp-EVMs suspension.

**Supplemental Table 1**

**Supplemental Table 1:** Primer Sequences of RT-qPCR

| **Gene** |  | **Sequence (5′ to 3′)** |
| --- | --- | --- |
| *TNF-α* | F: | CCCTCACACTCAGATCATCTTCT |
|  | R: | GCTACGACGTGGGCTACAG |
| *IL-6* | F: | TAGTCCTTCCTACCCCAATTTCC |
|  | R: | TTGGTCCTTAGCCACTCCTTC |
| *INOS* | F: | GTTCTCAGCCCAACAATACAAGA |
|  | R: | GTGGACGGGTCGATGTCAC |
| *COX-2* | F: | TGAGCAACTATTCCAAACCAGC |
|  | R: | GCACGTAGTCTTCGATCACTATC |
| *IL-1β* | F: | GCAACTGTTCCTGAACTCAACT |
|  | R: | ATCTTTTGGGGTCCGTCAACT |
